# Comparative effectiveness of sotrovimab and molnupiravir for prevention of severe covid-19 outcomes in patients in the community: observational cohort study with the OpenSAFELY platform

**DOI:** 10.1136/bmj-2022-071932

**Published:** 2022-11-16

**Authors:** Bang Zheng, Amelia C A Green, John Tazare, Helen J Curtis, Louis Fisher, Linda Nab, Anna Schultze, Viyaasan Mahalingasivam, Edward P K Parker, William J Hulme, Sebastian C J Bacon, Nicholas J DeVito, Christopher Bates, David Evans, Peter Inglesby, Henry Drysdale, Simon Davy, Jonathan Cockburn, Caroline E Morton, George Hickman, Tom Ward, Rebecca M Smith, John Parry, Frank Hester, Sam Harper, Amir Mehrkar, Rosalind M Eggo, Alex J Walker, Stephen J W Evans, Ian J Douglas, Brian MacKenna, Ben Goldacre, Laurie A Tomlinson

**Affiliations:** 1 London School of Hygiene and Tropical Medicine, London, UK; 2 The DataLab, Nuffield Department of Primary Care Health Sciences, University of Oxford, Oxford, UK; 3 TPP, TPP House, Horsforth, Leeds, UK

## Abstract

**Objective:**

To compare the effectiveness of sotrovimab (a neutralising monoclonal antibody) with molnupiravir (an antiviral) in preventing severe outcomes of covid-19 in adult patients infected with SARS-CoV-2 in the community and at high risk of severe outcomes from covid-19.

**Design:**

Observational cohort study with the OpenSAFELY platform.

**Setting:**

With the approval of NHS England, a real world cohort study was conducted with the OpenSAFELY-TPP platform (a secure, transparent, open source software platform for analysis of NHS electronic health records), and patient level electronic health record data were obtained from 24 million people registered with a general practice in England that uses TPP software. The primary care data were securely linked with data on SARS-CoV-2 infection and treatments, hospital admission, and death, over a period when both drug treatments were frequently prescribed in community settings.

**Participants:**

Adult patients with covid-19 in the community at high risk of severe outcomes from covid-19, treated with sotrovimab or molnupiravir from 16 December 2021.

**Interventions:**

Sotrovimab or molnupiravir given in the community by covid-19 medicine delivery units.

**Main outcome measures:**

Admission to hospital with covid-19 (ie, with covid-19 as the primary diagnosis) or death from covid-19 (ie, with covid-19 as the underlying or contributing cause of death) within 28 days of the start of treatment.

**Results:**

Between 16 December 2021 and 10 February 2022, 3331 and 2689 patients were treated with sotrovimab and molnupiravir, respectively, with no substantial differences in baseline characteristics. Mean age of all 6020 patients was 52 (standard deviation 16) years; 59% were women, 89% were white, and 88% had received three or more covid-19 vaccinations. Within 28 days of the start of treatment, 87 (1.4%) patients were admitted to hospital or died of infection from SARS-CoV-2 (32 treated with sotrovimab and 55 with molnupiravir). Cox proportional hazards models stratified by area showed that after adjusting for demographic information, high risk cohort categories, vaccination status, calendar time, body mass index, and other comorbidities, treatment with sotrovimab was associated with a substantially lower risk than treatment with molnupiravir (hazard ratio 0.54, 95% confidence interval 0.33 to 0.88, P=0.01). Consistent results were found from propensity score weighted Cox models (0.50, 0.31 to 0.81, P=0.005) and when restricted to people who were fully vaccinated (0.53, 0.31 to 0.90, P=0.02). No substantial effect modifications by other characteristics were detected (all P values for interaction >0.10). The findings were similar in an exploratory analysis of patients treated between 16 February and 1 May 2022 when omicron BA.2 was the predominant variant in England.

**Conclusions:**

In routine care of adult patients in England with covid-19 in the community, at high risk of severe outcomes from covid-19, those who received sotrovimab were at lower risk of severe outcomes of covid-19 than those treated with molnupiravir.

## Introduction

Neutralising monoclonal antibodies and antiviral medicines were approved by the UK Medicines and Healthcare products Regulatory Agency for use in patients with covid-19 not requiring admission to hospital to prevent progression of disease. On 16 December 2021, covid-19 medicine delivery units were launched across England to provide neutralising monoclonal antibodies and antivirals in community settings to treat patients with symptoms of covid-19 who were at high risk of severe outcomes.

Among the first available treatment options were sotrovimab (an intravenous neutralising monoclonal antibody) and molnupiravir (an oral antiviral).^1^ ^2^ ^3^ The approval and early clinical use of these drug treatments were mainly based on data from two phase 3 randomised controlled trials.^4^ ^5^ The findings of these trials could be limited, however, by the relatively small sample size, lack of population generalisability, given the strict inclusion and exclusion criteria, and the predominant circulating variants when the trials were conducted. In particular, little evidence is available on their effectiveness in patients with covid-19 who had received covid-19 vaccinations, in patients infected with omicron variants of the virus, or in those with severe renal or liver impairment. Uncertainty exists about the efficacy of molnupiravir in patients previously infected with SARS-CoV-2, in those with diabetes, and in non-white ethnic groups,^5^ and the appropriateness of early regulatory authorisation for this drug has been debated given the modest effect magnitude found in the randomised controlled trial.^6^ Also, both lower compliance with courses of oral drug treatment and longer time to administration of treatments after the onset of symptoms in routine care compared with clinical trials might affect potential benefit. Therefore, validating the effectiveness of sotrovimab and molnupiravir in preventing adverse outcomes in real world settings with varied populations is crucial in supporting their widescale clinical use in patients with covid-19.

In the first two months after the launch of covid-19 medicine delivery units, sotrovimab and molnupiravir were the most frequently prescribed drug treatments,^2^ with anecdotal reports that choice of drug was in part determined by the availability of facilities to deliver intravenous infusions and relative clinical equipoise for the choice of drug.^3^ This situation provided the opportunity for observational comparison of the effectiveness of the two drug treatments, possibly with limited bias according to patient characteristics. A comparative effectiveness study would also provide real world evidence for the clinical practice guideline on the prioritised treatment.^7^

Therefore, we sought to compare the effectiveness of sotrovimab versus molnupiravir in preventing severe outcomes from SARS-CoV-2 infection in adult patients in England with covid-19, who were at high risk of severe outcomes from infection but who did not require admission to hospital, during the first two months of the national rollout of covid-19 medicine delivery units, by using near real time electronic health record data in the OpenSAFELY-TPP platform. The OpenSAFELY-TPP platform is a secure, transparent, open source software platform for analysis of NHS electronic health records. We also explored the potential modifying effects of different demographic and clinical factors on the effectiveness of the drugs. We then conducted an exploratory analysis of patients treated in the next three months when omicron BA.2 had replaced BA.1 as the predominant variant of the SARS-CoV-2 virus in England.^8^

## Methods

### Study design and population

**Fig 1:**
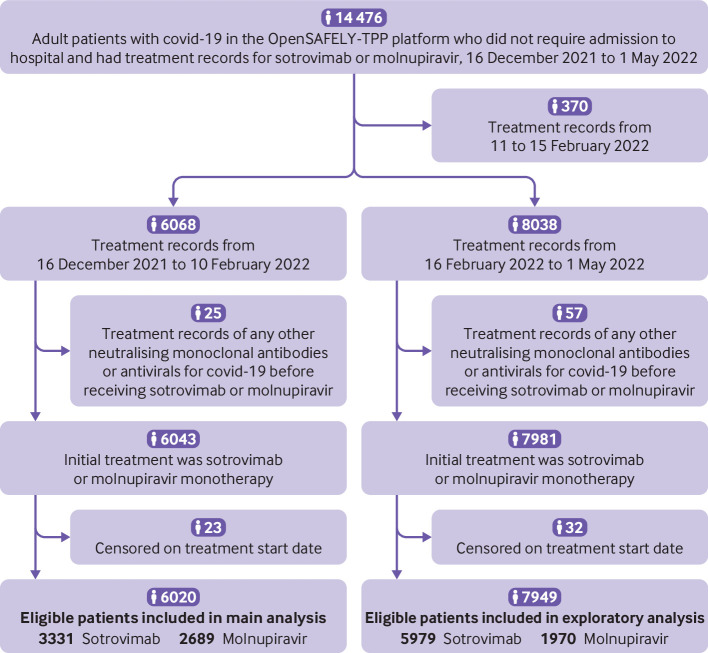
Study population flowchart. Patients treated before 1 May 2022 were included to allow sufficient follow-up time and time for linkage data to be updated in OpenSAFELY-TPP platform

In this observational cohort study, adult patients (aged ≥18 years) in the OpenSAFELY-TPP platform who had not required admission to hospital for covid-19 and had treatment records for sotrovimab or molnupiravir since 16 December 2021 in the covid-19 therapeutics dataset^2^ were included (fig 1). Our main analyses focused on those treated before 10 February 2022 (period 1) because after this date treatment recommendations were changed, with molnupiravir moved to third line treatment.^7^ We required patients to be registered at a general practice surgery at the start of treatment to allow us to extract baseline and follow-up information. According to the eligibility criteria from NHS England,^3^ to receive covid-19 neutralising monoclonal antibody or antiviral treatment in the community during this period, patients had to have SARS-CoV-2 infection confirmed by a positive polymerase chain reaction test result, have onset of covid-19 symptoms within the past five days, and belong to at least one of the following 10 high risk cohorts: Down’s syndrome, solid cancer, haematological disease or stem cell transplant, renal disease, liver disease, immune mediated inflammatory disorders, primary immune deficiencies, HIV/AIDS, solid organ transplant, or rare neurological conditions. Patients who had signs of recovery or required admission to hospital for covid-19 or supplemental oxygen specifically for the management of symptoms of covid-19 were not eligible to receive neutralising monoclonal antibody or antiviral treatment in the community.^3^ In the exploratory analysis (period 2), we included supplemental data from patients treated between 16 February (approximate date when the prevalence of the omicron BA.2 variant in England was >50%^8^) and 1 May 2022.

### Data sources

All data were linked, stored, and analysed securely within the OpenSAFELY platform (www.opensafely.org/). OpenSAFELY is a data analytics platform created by our team on behalf of NHS England to look at urgent covid-19 research questions. The dataset analysed in OpenSAFELY-TPP is based on 24 million people currently registered with GP surgeries that use TPP SystmOne software. Data include pseudonymised data, such as coded diagnoses, drug treatments, and physiological parameters. No free text data are included. All code is shared openly for review and re-use under Massachusetts Institute of Technology (MIT) open license (https://github.com/opensafely/sotrovimab-and-molnupiravir). Detailed pseudonymised patient data are potentially re-identifiable and therefore not shared. Primary care records managed by the GP software provider TPP are securely linked to other similarly pseudonymised datasets, including the Office for National Statistics mortality database, inpatient hospital records from the Secondary Uses Service, national coronavirus testing records from the Second Generation Surveillance System, and the covid-19 therapeutics dataset, a patient level dataset on neutralising monoclonal antibody and antiviral treatments derived from Blueteq software that covid-19 medicine delivery units use to notify NHS England of covid-19 treatments. Patient level vaccination status was available in the GP records directly from the National Immunisation Management System.

### Intervention

The intervention of interest was treatment with sotrovimab or molnupiravir given by covid-19 medicine delivery units, with the start date of treatment for each patient as recorded in the covid-19 therapeutics dataset. These two drugs were the only recommended treatments by NHS England from 16 December 2021 to 10 February 2022.^3^ Patients were excluded if they had treatment records of any other neutralising monoclonal antibodies or antiviral agents for covid-19 before receiving sotrovimab or molnupiravir (n=25 in period 1 and n=57 in period 2). Patients with treatment records of both sotrovimab and molnupiravir were censored at the start date of the second treatment (n=10 in period 1 and n=25 in period 2).

### Outcomes

The primary outcome was admission to hospital for covid-19 (ie, with covid-19 as the primary diagnosis from Secondary Uses Service) or death related to covid-19 (ie, with covid-19 as the underlying or contributing cause of death) within 28 days of the start of treatment. A diagnosis of covid-19 was recorded based on two ICD-10 (international classification of diseases,10th revision) codes (U07.1 and U07.2). Secondary outcomes were hospital admission or death from all causes within 28 days and hospital admission or death from covid-19 within 60 days of the start of treatment. To exclude events where patients were admitted to receive sotrovimab or other planned or regular treatment (eg, chemotherapy or dialysis), we did not count admissions coded as elective day case admission or regular admission in the Secondary Uses Service, or day cases detected by the same admission and discharge dates as outcome events (supplementary table 1 shows the breakdown). For patients who were admitted to hospital for covid-19, we also extracted information on admission to critical care units based on recorded days in critical care and procedure codes indicative of critical care.

### Covariates

Potential confounding factors or effect modifiers extracted at baseline were: age, sex, Sustainability Transformation Partnerships code of their registered GP surgery (an NHS administrative region assumed to be a proxy for covid-19 medicine delivery units), ethnic group (grouped into five broad categories: white, black or black British, Asian or Asian British, mixed, other), index of multiple deprivation (grouped into five categories derived from the patient’s postcode at lower super output area level to reflect socioeconomic status), rural-urban classification (derived from the patient’s postcode), calendar week (to account for secular trends in prescriptions and incidence of covid-19 outcomes), covid-19 vaccination status (unvaccinated, and one, two, or three or more vaccinations), date of SARS-CoV-2 infection positive test result (polymerase chain reaction or lateral flow test, as a proxy for the date of onset of symptoms), body mass index (the most recent record within 10 years: <18.5, 18.5-<25, 25-<30, and ≥30), 10 high risk cohort categories (allowing multiple categories for each patient), other comorbidities (diabetes, hypertension, chronic cardiac disease, chronic respiratory disease, dementia, autism, learning disabilities, and severe mental illness), and residency in a care home and housebound status. Detailed definitions and codelists for the covariates are available online (https://github.com/opensafely/sotrovimab-and-molnupiravir/tree/main/codelists). Individuals with missing information for ethnic group, index of multiple deprivation, rural-urban classification, body mass index, or positive SARS-CoV-2 test result were included as an unknown category for each variable.

### Statistical analyses

Distributions of baseline characteristics were compared for patients treated with sotrovimab versus molnupiravir with the t test, χ^2^ test, or rank sum test, where appropriate. Follow-up time for individual patients was calculated from the start date of the treatment record until the outcome event date, 28 days after the start of treatment, start of a second treatment with neutralising monoclonal antibody or antiviral agent, death, patient deregistration date, or the study end date (10 August 2022), whichever occurred first.

The risk of admission to hospital or death from covid-19 within 28 days in the two drug groups in period 1 were compared with Cox proportional hazards models, with time since treatment as the time scale. The Cox models were stratified by Sustainability Transformation Partnerships areas to account for geographic heterogeneity in baseline hazards, with sequential adjustment for other baseline covariates. Model 1 was adjusted for age and sex; model 2 was also adjusted for the 10 high risk cohort categories; model 3 was further adjusted for ethnic group, index of multiple deprivation (five categories), vaccination status, and calendar week; and model 4 was further adjusted for body mass index category, diabetes, hypertension, and chronic cardiac and respiratory diseases. The proportional hazards assumption was assessed by testing for a zero slope in the scaled Schoenfeld residuals for each Cox model.

We then adopted the propensity score weighting method as an alternative approach to account for confounding bias.^9^ We used propensity score weighting to balance the distributions of relevant covariates between the two drug groups. The propensity score for each patient was defined as the conditional probability of being treated with sotrovimab, estimated with a binary logistic regression of the actual treatment allocation on relevant baseline covariates. The average treatment effect weighting scheme was then applied to the Cox model based on the estimated propensity scores. Balance check of baseline covariates after weighting was conducted with standardised mean differences between groups (with a threshold of <0.10 as the indicator of well balanced). Robust variance estimators were used in the weighted Cox models. Missing values for covariates were treated as separate categories in the main analyses.

Similar analytical procedures were used for comparing risks of secondary outcomes between the groups. We also explored whether the following factors could modify the observed comparative effectiveness: each high risk cohort, covid-19 vaccination status (≥3 *v* <3), body mass index categories (≥30 *v* <30), presence of diabetes, hypertension, chronic cardiac diseases or chronic respiratory diseases, days between a positive test result and the start of treatment (<3 *v* 3-5 days), age group (<60 *v* ≥60 years), sex, and ethnic group (white *v* non-white). We tested effect modification by each of these variables by adding the corresponding interaction term between the variable and drug group in the stratified Cox model.

Sensitivity analyses based on the stratified Cox model were conducted to assess the robustness of the main findings, including: with complete case analysis or Multiple Imputation by Chained Equations for missing values (given the assumption of missing at random) instead of treating missing values as a separate category; with Cox models stratified by calendar week to account for potential temporal heterogeneity in baseline hazards, with conventional adjustment for other covariates; also adjusting for time between a positive test result and the start of treatment, and time between date of last vaccination and start of treatment; also adjusting for rural-urban classification, and other comorbidities and factors that might have influenced the clinician’s choice of treatment through the patient’s ability to travel to hospital for an infusion (dementia, autism, learning disabilities, severe mental illness, residency in a care home, or housebound status); with restricted cubic splines for age to further control for potential non-linear age effect; excluding patients with treatment records for both sotrovimab and molnupiravir, or with treatment records for any other treatment (ie, casirivimab, Paxlovid (combination of nirmatrelvir and ritonavir), or remdesivir); excluding patients who did not have a positive SARS-CoV-2 test record before treatment or started treatment after five days since a positive SARS-CoV-2 test result; creating a one day or two day lag in the follow-up start date to account for potential delays in drug administration (ie, start the follow-up on the second or third day after the recorded treatment date); applying a more strict definition of death related to covid-19 which requires covid-19 to be listed as the underlying cause of death; and conducting a competing risk analysis with admission to hospital or death from covid-19 and admission to hospital or death from other causes within 28 days as competing outcome events with the Fine-Gray subdistribution hazard model. Finally, to assess whether the main findings during period 1 when the omicron BA.1 was the predominant variant in England (December 2021-February 2022)^8^ persisted when BA.2 was the predominant variant, we conducted an exploratory analysis with data from patients treated during period 2, following similar analytical approaches.

### Software and reproducibility

Data management was performed with Python, with analysis carried out with Stata 16.1. Code for data management and analysis, as well as codelists, are archived online (https://github.com/opensafely/sotrovimab-and-molnupiravir). All iterations of the prespecified study protocol are archived with version control (https://github.com/opensafely/sotrovimab-and-molnupiravir/tree/main/docs).

### Patient and public involvement

Neither patients nor the public were involved in developing the research question and study, or in the design, management, or interpretation of this study. The primary barrier was the rapid timescale of analysis to deliver timely results. 

## Results

### Patient characteristics

Between 16 December 2021 and 10 February 2022, 6020 patients with covid-19 in the community in the OpenSAFELY-TPP platform and who met the study criteria were treated with sotrovimab (n=3331) or molnupiravir (n=2689) (fig 1). Mean age of the 6020 patients was 52.3 (standard deviation 16.0) years; 58.8% were women, 88.7% were white, and 87.6% had three or more covid-19 vaccinations. Compared with patients treated with molnupiravir, the sotrovimab group were slightly younger (mean age 51.7 *v* 52.9 years), and had a lower proportion of patients with Down’s syndrome (1.3% *v* 3.5%), immunosuppression (17.6% *v* 20.5%), and HIV/AIDS (2.2% *v* 4.4%). In contrast, we found a higher proportion of patients with renal disease (15.3% *v* 9.9%), solid organ transplant recipients (15.1% *v* 11.3%), and patients with obesity (36.5% *v* 34.4%) in the sotrovimab group than in the molnupiravir group. The two groups were similar for a wide range of other characteristics (table 1).

**Table 1:** Baseline characteristics of patients with covid-19 treated with molnupiravir or sotrovimab

Characteristics	Molnupiravir group (n=2689)	Sotrovimab group (n=3331)	Total (n=6020)
Age (years, mean (SD))*	52.9 (16.7)	51.7 (15.4)	52.3 (16.0)
Women	1551 (57.7)	1991 (59.8)	3542 (58.8)
White ethnic group	2348 (88.5)	2932 (88.9)	5280 (88.7)
Most deprived	372 (14.2)	486 (15.0)	858 (14.7)
Region (NHS)*:			
East	882 (32.8)	997 (29.9)	1879 (31.2)
London	245 (9.1)	171 (5.1)	416 (6.9)
East Midlands	293 (10.9)	673 (20.2)	966 (16.0)
West Midlands	46 (1.7)	186 (5.6)	232 (3.9)
North East	83 (3.1)	237 (7.1)	320 (5.3)
North West	291 (10.8)	284 (8.5)	575 (9.6)
South East	170 (6.3)	204 (6.1)	374 (6.2)
South West	454 (16.9)	402 (12.1)	856 (14.2)
Yorkshire	225 (8.4)	177 (5.3)	402 (6.7)
High risk cohorts:			
Down’s syndrome*	93 (3.5)	44 (1.3)	137 (2.3)
Solid cancer	387 (14.4)	528 (15.9)	915 (15.2)
Haematological disease	383 (14.2)	513 (15.4)	896 (14.9)
Renal disease*	266 (9.9)	509 (15.3)	775 (12.9)
Liver disease	142 (5.3)	159 (4.8)	301 (5.0)
Immune mediated inflammatory diseases	1261 (46.9)	1560 (46.8)	2821 (46.9)
Immunosuppression*	552 (20.5)	586 (17.6)	1138 (18.9)
HIV/AIDS*	118 (4.4)	73 (2.2)	191 (3.2)
Solid organ transplant*	303 (11.3)	504 (15.1)	807 (13.4)
Rare neurological disease	415 (15.4)	455 (13.7)	870 (14.5)
Body mass index (mean (SD))*	28.5 (6.4)	28.9 (6.6)	28.7 (6.6)
Diabetes	529 (19.7)	704 (21.1)	1233 (20.5)
Chronic cardiac disease	320 (11.9)	451 (13.5)	771 (12.8)
Hypertension	977 (36.3)	1270 (38.1)	2247 (37.3)
Chronic respiratory disease	547 (20.3)	652 (19.6)	1199 (19.9)
Vaccination status:			
None	71 (2.6)	63 (1.9)	134 (2.2)
One vaccination	49 (1.8)	58 (1.7)	107 (1.8)
Two vaccinations	243 (9.0)	265 (8.0)	508 (8.4)
Three or more vaccinations	2326 (86.5)	2945 (88.4)	5271 (87.6)
Time between positive test result and treatment (days, median (IQR))*	2 (2 to 3)	3 (2 to 3)	2 (2 to 3)
Time between start of campaign and treatment (weeks, median (IQR))*	4 (3 to 6)	5 (3 to 7)	5 (3 to 7)

Data are number (%) unless indicated otherwise.

SD=standard deviation; IQR=interquartile range.

Comparisons between groups were conducted with t test, χ^2^ test, or rank sum test, where appropriate: *P<0.05.

Ethnic group, index of multiple deprivation, body mass index, and date of positive test result had 68, 178, 598, and 231 missing values, respectively.

### Comparative effectiveness for the primary outcome

**Fig 2:**
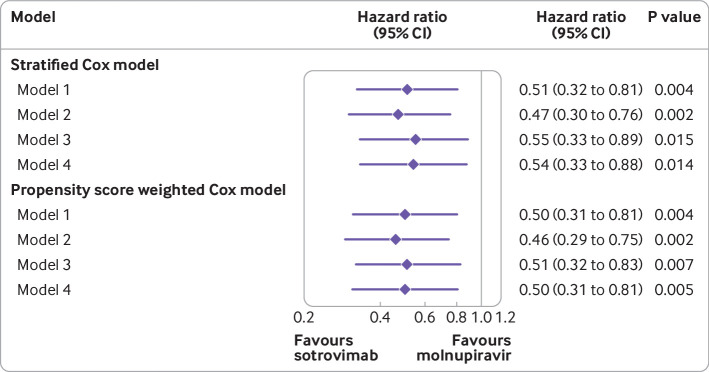
Comparing risk of admission to hospital or death from covid-19 during the 28 days of follow-up between patients treated with sotrovimab versus molnupiravir. Hazard ratio (95% confidence interval) for admission to hospital for covid-19 or death from covid-19. Model 1 adjusted for age and sex; model 2 also adjusted for 10 high risk cohort categories; model 3 further adjusted for ethnic group, index of multiple deprivation (five categories), vaccination status, and calendar week; and model 4 further adjusted for body mass index category, diabetes, hypertension, and chronic cardiac and respiratory diseases

Among the 6020 patients treated with sotrovimab or molnupiravir, 87 (1.45%) were admitted to hospital or died from covid-19 during the 28 days of follow-up after the start of treatment; 32 (0.96%) in the sotrovimab group and 55 (2.05%) in the molnupiravir group. Of these 87 patients, 25 (0.42%) died of covid-19 during the 28 days of follow-up (seven in the sotrovimab group and 18 in the molnupiravir group), among whom 16 died after admission to hospital for covid-19 and nine died in the community. The underlying cause of death was recorded as covid-19 for 20 of the 25 patients. Among the 78 patients who were admitted to hospital for covid-19, less than five of the 29 in the sotrovimab group and 11 of 49 in the molnupiravir group were recorded as having received critical care, and median duration in hospital was 6 (interquartile range 2-18) days for the sotrovimab group and 7.5 (4-12) days for the molnupiravir group. The results of the stratified Cox regression showed that after adjusting for demographic variables, the 10 high risk cohort categories, vaccination status, calendar week, body mass index category, and other comorbidities, treatment with sotrovimab was associated with a substantially lower risk of admission to hospital or death from covid-19 during the 28 days of follow-up than treatment with molnupiravir (hazard ratio 0.54, 95% confidence interval 0.33 to 0.88, P=0.01). Consistent results favouring sotrovimab over molnupiravir were obtained from propensity score weighted Cox models (model 4: hazard ratio 0.50, 95% confidence interval 0.31 to 0.81, P=0.005) after confirmation of successful balance of baseline covariates between groups in the weighted sample (supplementary fig 1). The magnitude of the hazard ratios was stable during the sequential covariate adjustment process (ranging from 0.46 to 0.55 across different models, fig 2). No violation of the proportional hazards assumption was detected in any model (P>0.10).

### Comparative effectiveness for secondary outcomes

For the secondary outcomes, 95 patients (1.58%) were admitted to hospital or died from covid-19 during the 60 days of follow-up after the start of treatment (34 in the sotrovimab group and 61 in the molnupiravir group). The results of stratified Cox regression showed a significantly lower risk in the sotrovimab group than in the molnupiravir group (hazard ratios ranging from 0.46 to 0.51 in models 1-4, all P<0.05, table 2). During the 28 days of follow-up after the start of treatment, 250 patients (4.17%) were admitted to hospital or died from any cause, 127 (3.83%) in the sotrovimab group and 123 (4.58%) in the molnupiravir group. Unlike the outcomes related to covid-19, we found no significant difference in the risk of admission to hospital or death from all causes between the sotrovimab group and the molnupiravir group in the stratified Cox regressions (hazard ratios ranging from 0.84 to 0.96 in models 1-4, all P>0.05, table 2).

**Table 2:** Comparison of risks of primary and secondary outcomes between patients treated with sotrovimab versus molnupiravir

Outcomes	No of patients	No of events	Hazard ratio (95% CI) for sotrovimab (reference=molnupiravir)
28 day follow-up: admission to hospital or death from covid-19 (sotrovimab/molnupiravir)	6020 (3331/2689)	87 (32/55)	—
Model 1	—	—	0.51 (0.32 to 0.81)
Model 2	—	—	0.47 (0.30 to 0.76)
Model 3	—	—	0.55 (0.33 to 0.89)
Model 4	—	—	0.54 (0.33 to 0.88)
60 day follow-up: admission to hospital or death from covid-19 (sotrovimab/molnupiravir)	6020 (3331/2689)	95 (34/61)	—
Model 1	—	—	0.50 (0.32 to 0.79)
Model 2	—	—	0.46 (0.29 to 0.73)
Model 3	—	—	0.51 (0.32 to 0.82)
Model 4	—	—	0.50 (0.31 to 0.81)
28 day follow-up: admission to hospital or death for all causes (sotrovimab/molnupiravir)	6001 (3318/2683)	250 (127/123)	—
Model 1	—	—	0.96 (0.73 to 1.26)
Model 2	—	—	0.84 (0.64 to 1.10)
Model 3	—	—	0.87 (0.66 to 1.16)
Model 4	—	—	0.86 (0.65 to 1.14)

CI=confidence interval. Results based on Cox model stratified by area. Crude hazard ratios (95% confidence intervals) were 0.47 (0.30 to 0.72), 0.45 (0.29 to 0.68), and 0.83 (0.65 to 1.06) for the three outcomes, respectively.

Model 1 adjusted for age and sex; model 2 also adjusted for 10 high risk cohort categories; model 3 further adjusted for ethnic group, index of multiple deprivation (five categories), vaccination status, and calendar week; model 4 further adjusted for body mass index category, diabetes, hypertension, and chronic cardiac and respiratory diseases.

### Sensitivity analyses and tests for effect modification

The results of the sensitivity analyses were consistent with the main findings (hazard ratios for the primary outcome ranging from 0.51 to 0.58 across different analyses, table 3). Among patients included in the competing risk analysis (n=6001), 80 were admitted to hospital or died from covid-19 and 170 were admitted to hospital or died from other causes within 28 days of the start of treatment. The cause specific Cox model showed that sotrovimab was associated with a lower risk of admission to hospital or death from covid-19 (model 4: hazard ratio 0.51, 95% confidence interval 0.31 to 0.86, P=0.01) but had no significant association with admission to hospital or death from other causes compared with molnupiravir (model 4: 1.13, 0.80 to 1.59, P=0.48). Similarly, the Fine-Gray subdistribution hazard model showed that compared with molnupiravir, sotrovimab was associated with a lower cumulative incidence of admission to hospital or death from covid-19 (subdistribution hazard ratio 0.50, 95% confidence interval 0.30 to 0.85, P=0.01) but not for admission to hospital or death from other causes (1.15, 0.82 to 1.62, P=0.42).

**Table 3:** Sensitivity analyses for risk of admission to hospital or death from covid-19 during the 28 days of follow-up after the start of treatment with sotrovimab versus molnupiravir

Sensitivity analyses	No of patients	No of events	Hazard ratio (95% CI) for sotrovimab (reference=molnupiravir)
Main analysis (for comparison purpose) (sotrovimab/molnupiravir)	6020 (3331/2689)	87 (32/55)	0.54 (0.33 to 0.88)
Complete case analysis (sotrovimab/molnupiravir)	5214 (2889/2325)	74 (26/48)	0.53 (0.31 to 0.91)
Multiple imputation for missing values in covariates*	6020	87	0.54 (0.33 to 0.89)
With Cox models stratified by calendar week*	6020	87	0.53 (0.33 to 0.88)
Further adjusted for time between positive test result or last vaccination date and start of treatment*	6020	87	0.52 (0.32 to 0.86)
Further adjusted for rural-urban classification, other comorbidities, residency in care home, and housebound status*	6020	87	0.55 (0.33 to 0.90)
With restricted cubic splines to control for age effect*	6020	87	0.52 (0.32 to 0.86)
Excluding patients with treatment records of both sotrovimab and molnupiravir, or with any other treatments†	6010	87	0.54 (0.33 to 0.88)
Excluding patients without positive test record before treatment or started treatment after 5 days since positive test result†	5725	82	0.56 (0.33 to 0.93)
Creating one day lag in follow-up start date (sotrovimab/molnupiravir)	6001 (3324/2677)	74 (27/47)	0.51 (0.30 to 0.87)
Creating two day lag in follow-up start date (sotrovimab/molnupiravir)	5985 (3318/2667)	63 (25/38)	0.57 (0.32 to 1.02)
Defining death from covid-19 based on underlying cause of death alone†	6020‡	85‡	0.58 (0.35 to 0.96)

CI=confidence interval. Sensitivity analyses were based on fully adjusted stratified Cox model (model 4).

* Group specific counts in these analyses were the same as the main analysis.

† Group specific counts in these analyses were redacted to avoid inadvertent disclosure of small event numbers.

‡ Rounded to the nearest five to avoid inadvertent disclosure of small event numbers.

No substantial effect modification was seen for any of the 10 high risk cohort categories, covid-19 vaccination status, presence of obesity, diabetes, hypertension, chronic cardiac diseases or chronic respiratory diseases, time since positive test result, age group, sex, or ethnic group (P for interaction >0.10, supplementary fig 2). We found similar results to the main analysis in the subset of 5271 patients who had three or more covid-19 vaccinations (model 4 for the primary outcome: hazard ratio 0.53 for sotrovimab *v* molnupiravir, 95% confidence interval 0.31 to 0.90, P=0.02).

### Exploratory analyses of comparative effectiveness when omicron BA.2 was the predominant variant

A further 7949 patients with covid-19 treated with sotrovimab (n=5979) or molnupiravir (n=1970) between 16 February and 1 May 2022 were included in the exploratory analysis (fig 1). Patients included during period 2 were older (mean age 58.8, standard deviation 16.0) and had a higher proportion of white people (95.2%) and people who were fully vaccinated (94.0%) than those treated during period 1. Compared with patients treated with molnupiravir, those in the sotrovimab group were younger (mean age 57.9 *v* 61.4 years), and had a lower proportion of patients with Down’s syndrome (1.4% *v* 3.4%), immune mediated inflammatory disorders (44.7% *v* 47.7%), diabetes (22.7% *v* 25.3%), chronic cardiac diseases (17.9% *v* 21.5%), and hypertension (46.9% vs. 50.8%), and a higher proportion of solid organ transplant recipients (15.0% *v* 11.0%) and patients with haematological disease (18.3% *v* 14.9%). The two groups were similar for other characteristics (supplementary table 2).

Among the 7949 patients, 97 (1.22%) were admitted to hospital or died from covid-19 during the 28 days of follow-up; 57 (0.95%) in the sotrovimab group and 40 (2.03%) in the molnupiravir group. Of these 97 patients, 28 (0.35%) died of covid-19 during the 28 days of follow-up (nine in the sotrovimab group and 19 in the molnupiravir group). Treatment with sotrovimab was associated with a substantially lower risk of admission to hospital or death from covid-19 during the 28 days of follow-up than treatment with molnupiravir in the stratified Cox regression (model 4: hazard ratio 0.44, 95% confidence interval 0.27 to 0.71, P=0.001) and propensity score weighted Cox model (model 4: 0.53, 0.32 to 0.86, P=0.010) (supplementary fig 3 and fig 4). The magnitude of the hazard ratios was stable during the sequential covariate adjustment process (ranging from 0.43 to 0.55 across different models, supplementary fig 3). No violation of the proportional hazards assumption was detected in any model (P>0.10).

## Discussion

### Principal findings

In this national, real world cohort study, we assessed the comparative effectiveness of sotrovimab and molnupiravir in preventing severe covid-19 outcomes in patients with covid-19 who did not require admission to hospital. We used the multi-sourced electronic health record data in the OpenSAFELY-TPP platform to provide timely evidence to guide the clinical management of covid-19. We focused on patients treated between 16 December 2021 and 10 February 2022 in the main analysis to ensure that the two drug groups were comparable and to reduce confounding by indication based on the clinical guidelines at that time.^3^ The results showed a consistent and robust effect estimate of a lower risk of admission to hospital or death from covid-19 among those treated with sotrovimab compared with molnupiravir after applying different analytical approaches and when adjusting for a wide range of potential confounders, and in subgroup analyses including those underrepresented in clinical trials. The results were consistent with the exploratory analysis of patients receiving treatments between 16 February and 1 May 2022 when omicron BA.2 was the predominant variant in England.

### Findings in context

Our findings are in line with published trial results even though our study was conducted when the omicron variant was the predominant variant of the virus. The COMET-ICE (Covid-19 Monoclonal Antibody Efficacy Trial-Intent to Care Early) trial^4^ was a phase 3, double blind, randomised controlled trial that evaluated the use of sotrovimab in high risk adult patients in the community with symptoms of covid-19 who had not been vaccinated. An interim analysis of 583 patients from four countries showed a reduced risk of admission to hospital or death from all causes within 28 days in the sotrovimab group compared with the placebo group (1% *v* 7%, P=0.002).^4^ Similar results were reported for the final sample of 1057 patients from five countries, with a risk estimate of 1% with sotrovimab versus 6% with placebo (adjusted relative risk 0.21, absolute risk difference −4.53%, 95% confidence interval −6.70% to −2.37%, P<0.001).^10^ In contrast, a weaker effect was found in the phase 3 component of the MOVe-OUT (Efficacy and Safety of Molnupiravir (MK-4482) in Non-Hospitalized Adult Participants With COVID-19 (MK-4482-002)) trial^5^ for molnupiravir. MOVe-OUT was also a double blind, randomised controlled trial in adults in the community with mild to moderate covid-19 who were not vaccinated and had at least one risk factor for severe illness. The interim results of 775 participants from 15 countries showed that the risk of admission to hospital or death from all causes during the 28 day follow-up was lower with molnupiravir than with placebo (7.3% *v* 14.1%, absolute risk difference −6.8%, 95% confidence interval −11.3% to −2.4%, P=0.001).^5^ In the final sample of 1433 participants from 20 countries, however, a lower efficacy was seen, with the risk estimate of 6.8% in the molnupiravir group versus 9.7% in the placebo group (relative risk 0.70, absolute risk difference −3.0%, 95% confidence interval −5.9% to −0.1%, P=0.04).^5^

Evidence from several in vitro or in vivo studies indicated that both sotrovimab and molnupiravir were active against the omicron BA.1 variant (the predominant variant during the treatment period in our main analysis^8^).^11^ ^12^ ^13^ ^14^ Concerns have been raised about the possible loss of efficacy of sotrovimab against the omicron BA.2 variant, however, and the US National Institutes of Health no longer recommends sotrovimab for covid-19 treatment for this reason.^15^ Nevertheless, the existing evidence has been contradictory. For example, the omicron BA.2 sublineage had marked resistance to sotrovimab in in vitro experiments,^16^ ^17^ and a recent in vitro study reported similar findings for the BA.2.12.1, BA.4, and BA.5 sublineages.^18^ In contrast, an in vivo experiment found that both molnupiravir and sotrovimab can restrict viral replication in the lungs of hamsters infected with BA.2.^19^ Our exploratory analysis, conducted during the period when BA.2 was the predominant strain, supported the persistent protective role of sotrovimab against this subvariant. This finding was also in line with preliminary epidemiological data from the UK Health Security Agency that the risk of admission to hospital after sotrovimab use was similar in the periods when the predominant variants were BA.1 and BA.2.^20^

### Policy implications and interpretation

The current clinical guideline from NHS England 7 has de-prioritised molnupiravir for routine clinical use in adult patients with symptoms of covid-19 in the community at high risk of severe outcomes from covid-19, based on the results of recent trials.^5^ Sotrovimab is recommended as one of the first line treatment options (along with Paxlovid), whereas molnupiravir is considered a third line option (after a second line antiviral, remdesivir), and is only recommended when the other drugs cannot be used because of contraindications or feasibility issues. However, no comparative effectiveness trial has been conducted to support these clinical pathways. Our real world findings during a period when both drugs were frequently prescribed provide supportive evidence for this updated guideline. Assuming that molnupiravir had limited or no effect on covid-19 outcomes, our results imply that sotrovimab substantially reduced the risk of admission to hospital or death from covid-19 compared with eligible patients who did not receive sotrovimab or other drugs in real world settings.

The COMET-ICE and MOVe-OUT trials recruited only patients who were not vaccinated, and uncertainty has been raised about the effects in populations who have received covid-19 vaccinations.^3^ ^21^ This issue is a concern for both sotrovimab (whether active immunity induced by the vaccine influences passive immunisation with neutralising monoclonal antibodies 22) and molnupiravir (given the preliminary finding of limited efficacy in seropositive patients from the MOVe-OUT trial 5). Our analysis restricted to patients who had received three or more covid-19 vaccinations supports the conclusion that sotrovimab is beneficial in patients who are fully vaccinated, who now represent most of the covid-19 patient population in many settings.^23^

### Strengths and weaknesses

The key strengths of our study were the scale, level of detail, and completeness of the underlying primary care electronic health record data, and linkage to multiple covid-19 relevant national databases within the OpenSAFELY-TPP platform. Also, direct comparisons of the effectiveness of sotrovimab and molnupiravir were possible because of the concurrent national rollout of the two drugs under similar indications between 16 December 2021 and 10 February 2022.

Several limitations of the study need to be considered. Patients included in our study were assumed to be only those who met the eligibility criteria of NHS England,^3^ thus limiting further generalisation of our findings to people not at high risk of severe outcomes from covid-19. Also, because the information on admission to hospital or death from covid-19 was extracted from hospital records and death certificates, misclassification bias in outcome events could have been present, including uncertainty about whether patients were admitted to hospital or died from covid-19 or from a different cause while infected with SARS-CoV-2. Nevertheless, the different results for admission to hospital or death from covid-19 or from all causes and the cause specific analyses provided indirect evidence for the accuracy of the primary outcome. We defined admission to hospital for covid-19 based on the primary diagnosis code in the hospital records, which could largely reflect the primary reason for admission to hospital. The possibility of residual confounding cannot be ruled out in this real world observational study, in particular related to differences in the severity of covid-19 or other unmeasured clinical factors that might have influenced the clinician’s choice of treatment at the first assessment. This potential confounding bias could be more evident in the exploratory analyses after February 2022, when clinical equipoise between prescribing guidelines for sotrovimab and molnupiravir no longer existed. Therefore, these findings should be interpreted with caution. Given the size of the observed effect and its robustness across multiple sensitivity analyses, however, such bias would have to be substantial to fully explain the findings. Finally, our results cannot be used to infer lack of efficacy of molnupiravir for use in community settings; results from large scale randomised controlled trials, such as the UK PANORAMIC trial (www.panoramictrial.org/), are needed to draw such causal conclusions.

### Future research

Despite the potential benefits of treatment options for patients with covid-19 in the community in preventing admission to hospital or death from covid-19, some safety concerns still need to be explored with real world data. Apart from mild or moderate symptoms after treatment reported during the trials, some uncommon side effects such as urticaria and anaphylaxis have been seen for sotrovimab,^24^ and a preclinical study of molnupiravir suggested a possibility of bone marrow suppression and thrombocytopenia.^25^ Immediate post-marketing surveillance, especially with large scale electronic health record data, is vital to comprehensively characterise and quantify the risk-benefit balance for these newly available drugs.

On the other hand, the lower baseline risk of severe outcomes^26^ ^27^ ^28^ ^29^ as a result of the current prevalence of omicron variants and high population rates of vaccination or previous infection, or both, could result in lower absolute risk reduction by these drug treatments. This situation might change with future variants, as might the effectiveness of neutralising monoclonal antibodies and antiviral agents. Cost effectiveness studies of administration of neutralising monoclonal antibodies and antiviral agents in patients with covid-19 in the community might also be informative,^23^ ^30^ especially for neutralising monoclonal antibodies because of the higher price and administration costs.

### Conclusion

Our findings suggest that in routine care, sotrovimab was associated with a substantially lower risk of severe outcomes of covid-19 compared with molnupiravir in adult patients in England with covid-19 at high risk of severe outcomes from infection but who did not require admission to hospital, including those who were fully vaccinated. This study shows that monitoring of drug effects early after implementation can be used to provide direct evidence to support treatment decisions, and the results are consistent with current UK guidelines favouring the use of sotrovimab over molnupiravir.

**What is already known on this topic**

Two phase 3 randomised controlled trials in patients with covid-19 in the community, who were not vaccinated and at high risk of severe outcomes from covid-19, showed strong efficacy for sotrovimab in preventing admission to hospital or death (relative risk reduction by 79%) and modest efficacy for molnupiravir (30%)
No randomised controlled trial comparing these drug treatments has been published, and evaluations of their effectiveness when used in routine care are limited
Whether the effectiveness of sotrovimab and molnupiravir persists in people who are vaccinated, in patients infected with omicron variants, and in other subgroups underrepresented in clinical trials is unclear

**What this study adds**

This real world cohort study showed that in the routine care of adult patients in England with covid-19 in the community, at high risk of severe outcomes from infection, those receiving sotrovimab had a substantially lower risk of severe covid-19 outcomes than those treated with molnupiravir when omicron BA.1 and BA.2 were the predominant variants
This study extends previous findings from randomised controlled trials to populations who were vaccinated and infected with omicron variants
The findings support the current clinical guideline which prioritises sotrovimab over molnupiravir in patients with covid-19 who do not require admission to hospital

## Data Availability

Access to the underlying identifiable and potentially re-identifiable pseudonymised electronic health record data are tightly governed by various legislative and regulatory frameworks, and restricted by best practice. The data in OpenSAFELY are drawn from general practice data across England where TPP is the data processor. TPP developers initiate an automated process to create pseudonymised records in the core OpenSAFELY database, which are copies of key structured data tables in the identifiable records. These pseudonymised records are linked onto key external data resources that have also been pseudonymised via SHA-512 one way hashing of NHS numbers with a shared salt. Bennett Institute for Applied Data Science developers and principal investigators holding contracts with NHS England have access to the OpenSAFELY pseudonymised data tables as needed to develop the OpenSAFELY tools. These tools in turn enable researchers with OpenSAFELY data access agreements to write and execute code for data management and data analysis without direct access to the underlying raw pseudonymised patient data, and to review the outputs of this code. All code for the full data management pipeline, from raw data to completed results for this analysis, and for the OpenSAFELY platform as a whole, is available for review at https://github.com/OpenSAFELY.

## Web extra

Extra material supplied by authors

Web appendix: Supplementary materials

## Notes

Contributors: LAT, BG, BZ, AJW, SJWE, IJD, and BM conceptualised and designed the study. BZ, ACAG, and JT contributed to the coding and statistical analysis. BZ and LAT drafted the original version of the manuscript. AM, BG, CB, JP, CEM, HJC, and SCJB contributed to information governance and project administration. All authors contributed to interpretation of the results and critical revision of the manuscript. All authors approved the final manuscript. LAT and BG are guarantors for this study. The corresponding author attests that all listed authors meet authorship criteria and that no others meeting the criteria have been omitted.

The lead authors (LAT and BZ) affirm that the manuscript is an honest, accurate, and transparent account of the study being reported; that no important aspects of the study have been omitted; and that any discrepancies from the study as planned (and, if relevant, registered) have been explained.

Dissemination to participants and related patient and public communities: The results of this research will be disseminated to the public through press releases and lay summaries on study websites. A publicly available website (https://www.opensafely.org/) has been developed through which any patient or member of the public is invited to contact the authors regarding this study or the broader OpenSAFELY project.

Provenance and peer review: Not commissioned; externally peer reviewed.
