# Supplementary material for: Comparative effectiveness of sotrovimab and molnupiravir for prevention of severe covid-19 outcomes in patients in the community: observational cohort study with the OpenSAFELY platform
Source: BMJ. 2022 Nov 16;379:e071932. doi: 10.1136/bmj-2022-071932 (PMC9667468; doi:10.1136/bmj-2022-071932)
Supplement: Supplementary file 1 — Web appendix: Supplementary materials [file zheb071932.ww.pdf]

Supplementary Materials

Supplementary Table 1. Detected day case admissions that were not counted as hospitalisation events.

Admissions of which the admission and discharge dates were the same	Number of events	
	**Molnupiravir**	**Sotrovimab**
**Period 1 (main analysis)**		
Detected day cases on Day 0 (i.e., the treatment start date)	46	294
Those with MAbs procedure on Day 0	9	267
Detected day cases on Day 1	<5	36
Those with MAbs procedure on Day 1	<5	26
Detected day cases on or after Day 2	<5	7
Those with MAbs procedure on or after Day 2	<5	6
**Period 2 (exploratory analysis)**		
Detected day cases on Day 0 (i.e., the treatment start date)	44	317
Those with MAbs procedure on Day 0	27	283
Detected day cases on Day 1	<5	39
Those with MAbs procedure on Day 1	<5	34
Detected day cases on or after Day 2	6	17
Those with MAbs procedure on or after Day 2	<5	13

Note: The following admissions were not counted as hospitalisation events: (1) those recorded as “elective day case admission” or “regular admission” in the patient classification field of SUS dataset, because such coding clearly indicates that the patient was admitted for a planned procedure or regular treatment (thus cannot represent severe COVID-19 outcomes); or (2) day cases that we additionally detected with the admission and discharge dates being the same. As shown in this table, most of those additionally detected day case admissions were for monoclonal antibody infusion procedure (i.e., admitted for receiving sotrovimab). Monoclonal antibody procedures were identified based on OPCS codes (X891, X892).

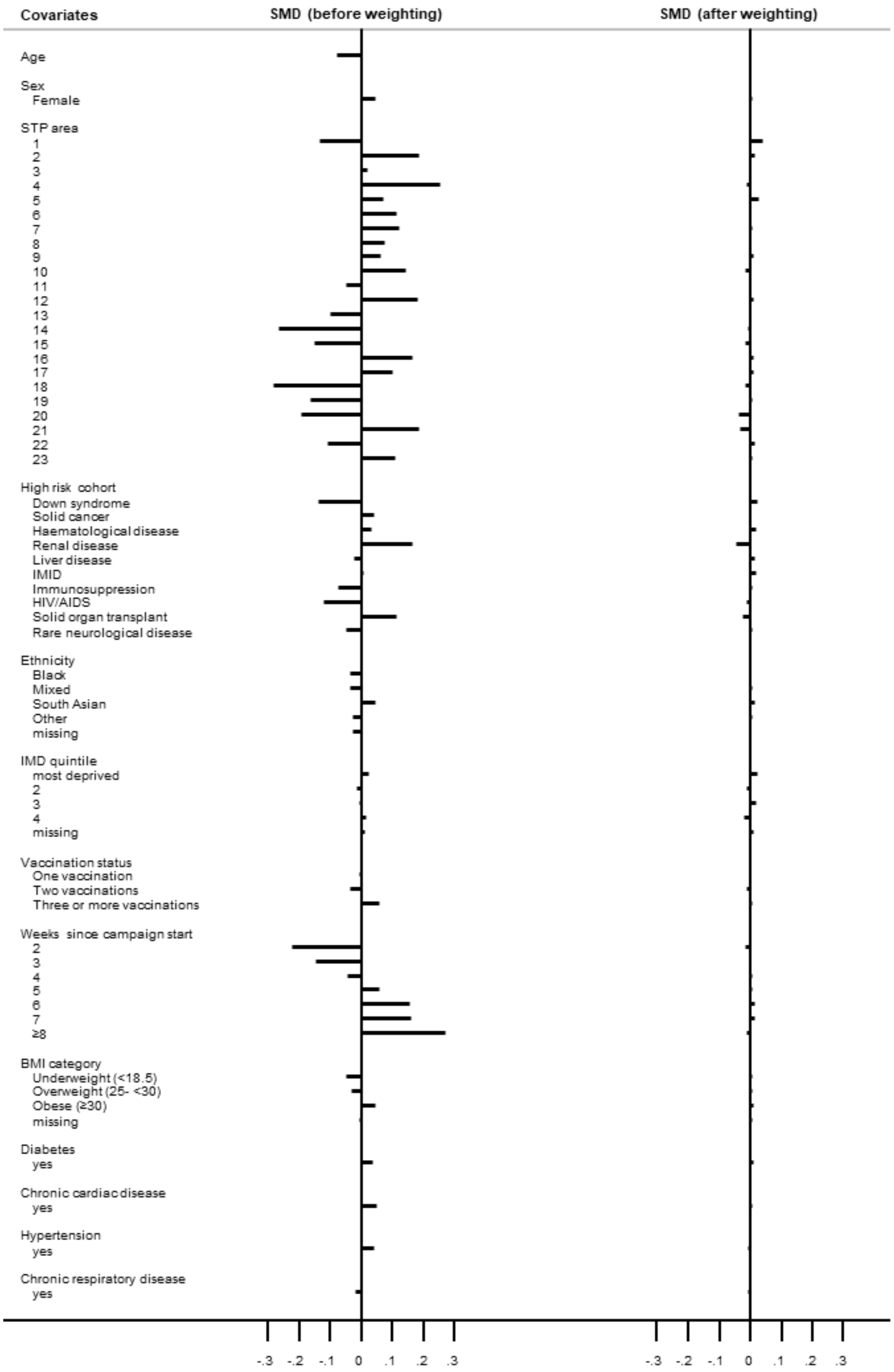

Supplementary Figure 1. Balance check of baseline covariates between treatment groups before and after propensity score weighting (Period 1).

Note: SMD=standardised mean difference; STP=Sustainability Transformation Partnerships; IMID=Immune-Mediated Inflammatory Diseases; IMD=Index of Multiple Deprivation; BMI=body mass index.

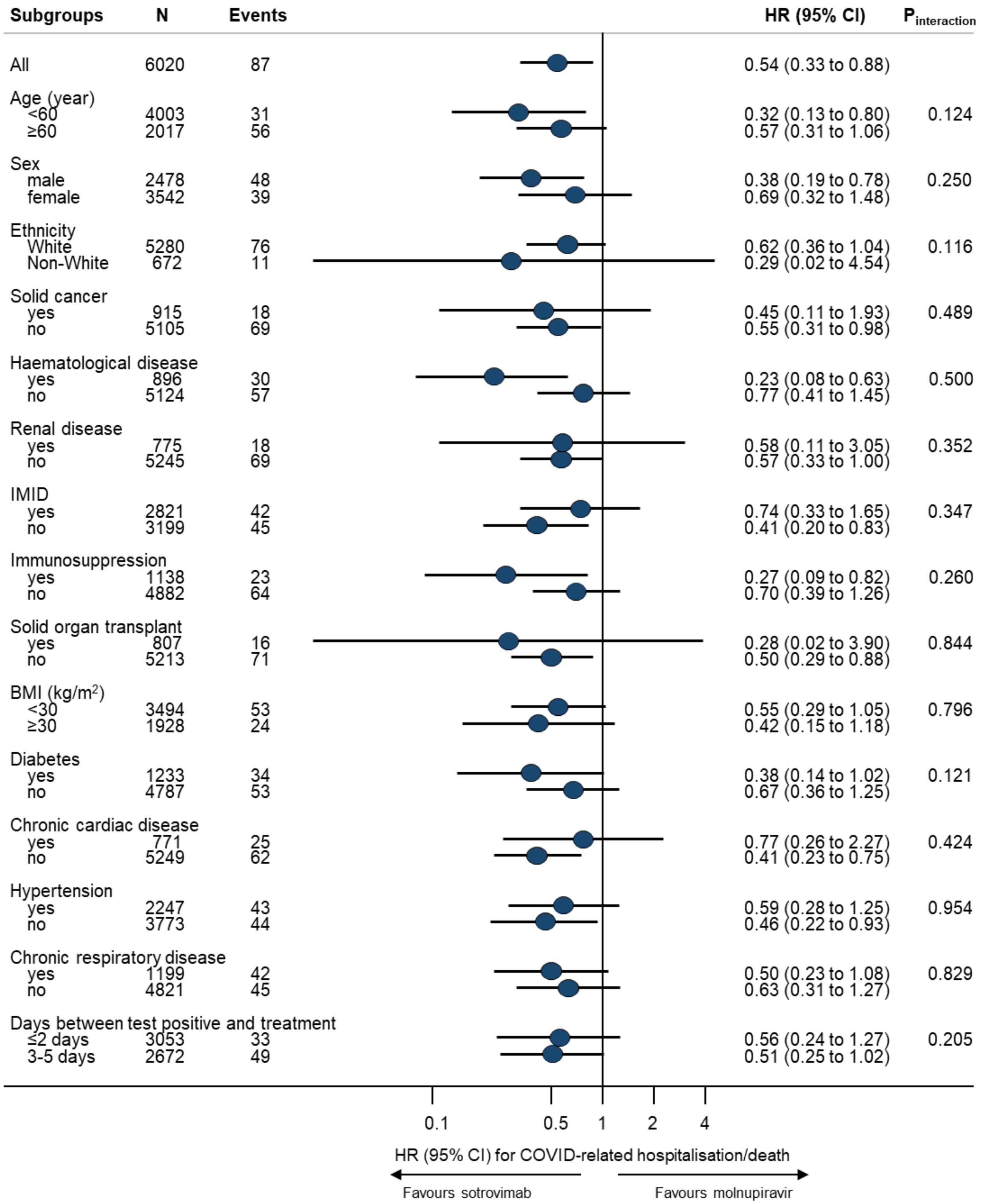
Subgroups N Events HR (95% CI) P interaction
All 6020 87 0.54 (0.33 to 0.88)
Age (year)
<60 4003 31 0.32 (0.13 to 0.80) 0.124
≥60 2017 56 0.57 (0.31 to 1.06)
Sex
male 2478 48 0.38 (0.19 to 0.78) 0.250
female 3542 39 0.69 (0.32 to 1.48)
Ethnicity
White 5280 76 0.62 (0.36 to 1.04) 0.116
Non-White 672 11 0.29 (0.02 to 4.54)
Solid cancer
yes 915 18 0.45 (0.11 to 1.93) 0.489
no 5105 69 0.55 (0.31 to 0.98)
Haematological disease
yes 896 30 0.23 (0.08 to 0.63) 0.500
no 5124 57 0.77 (0.41 to 1.45)
Renal disease
yes 775 18 0.58 (0.11 to 3.05) 0.352
no 5245 69 0.57 (0.33 to 1.00)
IMID
yes 2821 42 0.74 (0.33 to 1.65) 0.347
no 3199 45 0.41 (0.20 to 0.83)
Immunosuppression
yes 1138 23 0.27 (0.09 to 0.82) 0.260
no 4882 64 0.70 (0.39 to 1.26)
Solid organ transplant
yes 807 16 0.28 (0.02 to 3.90) 0.844
no 5213 71 0.50 (0.29 to 0.88)
BMI (kg/m2)
<30 3494 53 0.55 (0.29 to 1.05) 0.796
≥30 1928 24 0.42 (0.15 to 1.18)
Diabetes
yes 1233 34 0.38 (0.14 to 1.02) 0.121
no 4787 53 0.67 (0.36 to 1.25)
Chronic cardiac disease
yes 771 25 0.77 (0.26 to 2.27) 0.424
no 5249 62 0.41 (0.23 to 0.75)
Hypertension
yes 2247 43 0.59 (0.28 to 1.25) 0.954
no 3773 44 0.46 (0.22 to 0.93)
Chronic respiratory disease
yes 1199 42 0.50 (0.23 to 1.08) 0.829
no 4821 45 0.63 (0.31 to 1.27)
Days between test positive and treatment
≤2 days 3053 33 0.56 (0.24 to 1.27) 0.205
3-5 days 2672 49 0.51 (0.25 to 1.02)
0.1
0.5
1
2
4
HR (95% CI) for COVID-related hospitalisation/death
Favours sotrovimab
Favours molnupiravir

Supplementary Figure 2. Subgroup analysis of sotrovimab vs. molnupiravir in association with risk of 28-day COVID-19 related hospitalisation/death.

Note: HR=hazard ratio; CI=confidence interval; IMID=Immune-Mediated Inflammatory Diseases; BMI=body mass index. Subgroup analyses were based on the fully-adjusted stratified Cox model (Model 4). P for interaction between drug group and each of the following variables was: Down syndrome (0.221), liver disease (0.953), HIV/AIDS (0.999), rare neurological disease (0.999), vaccination status (0.574), respectively; no analyses within each level of these variables were done because of lack of sample size or outcome events within the subset of population.

Supplementary Table 2. Baseline characteristics of COVID-19 patients receiving molnupiravir or sotrovimab (Period 2).

Characteristics	Molnupiravir group	Sotrovimab group	Total
N	1970	5979	7949
Age (year), mean (SD)*	61.4 (16.6)	57.9 (15.6)	58.8 (16.0)
Female, n (%)	1112 (56.5)	3494 (58.4)	4606 (57.9)
White, n (%)	1863 (95.9)	5632 (95.0)	7495 (95.2)
Most deprived, n (%)	213 (11.0)	722 (12.4)	935 (12.0)
Region (NHS), n (%)*			
East	823 (41.8)	1885 (31.5)	2708 (34.1)
London	67 (3.4)	205 (3.4)	272 (3.4)
East Midlands	108 (5.5)	1355 (22.7)	1463 (18.4)
West Midlands	20 (1.0)	252 (4.2)	272 (3.4)
North East	17 (0.9)	240 (4.0)	257 (3.2)
North West	71 (3.6)	553 (9.2)	624 (7.9)
South East	149 (7.6)	360 (6.0)	509 (6.4)
South West	471 (23.9)	819 (13.7)	1290 (16.2)
Yorkshire	244 (12.4)	310 (5.2)	554 (7.0)
High-risk cohorts, n (%)			
Down syndrome*	66 (3.4)	84 (1.4)	150 (1.9)
Solid cancer	378 (19.2)	1071 (17.9)	1449 (18.2)
Haematological disease*	294 (14.9)	1094 (18.3)	1388 (17.5)
Renal disease	276 (14.0)	901 (15.1)	1177 (14.8)
Liver disease	105 (5.3)	323 (5.4)	428 (5.4)
Immune-mediated inflammatory diseases*	940 (47.7)	2673 (44.7)	3613 (45.5)
Immunosuppression	308 (15.6)	948 (15.9)	1256 (15.8)
HIV/AIDS	41 (2.1)	101 (1.7)	142 (1.8)
Solid organ transplant*	216 (11.0)	896 (15.0)	1112 (14.0)
Rare neurological disease	237 (12.0)	742 (12.4)	979 (12.3)
BMI (kg/m^2), mean (SD)	28.8 (6.3)	28.9 (6.6)	28.9 (6.5)
Diabetes, n (%)*	498 (25.3)	1359 (22.7)	1857 (23.4)
Chronic cardiac disease, n (%)*	424 (21.5)	1068 (17.9)	1492 (18.8)
Hypertension, n (%)*	1001 (50.8)	2806 (46.9)	3807 (47.9)
Chronic respiratory disease, n (%)	493 (25.0)	1394 (23.3)	1887 (23.7)
Vaccination status, n (%)			

Characteristics	Molnupiravir group	Sotrovimab group	Total
None	27 (1.4)	74 (1.2)	101 (1.3)
One vaccination	15 (0.8)	54 (0.9)	69 (0.9)
Two vaccinations	84 (4.3)	223 (3.7)	307 (3.9)
Three or more vaccinations	1844 (93.6)	5628 (94.1)	7472 (94.0)
Days between test positive and treatment, median (IQR)*	2 (1-3)	2 (1-3)	2 (1-3)
Weeks between campaign start and treatment, median (IQR)*	15 (13-17)	15 (13-17)	15 (13-17)

Note: SD=standard deviation; BMI=body mass index; IQR=interquartile range. Comparisons between groups were conducted using *t*-test, chi-square test or rank-sum test, where appropriate; * indicates P<0.05. Ethnicity, Index of Multiple Deprivation, BMI and positive test date had 79, 188, 633 and 982 missing values, respectively.

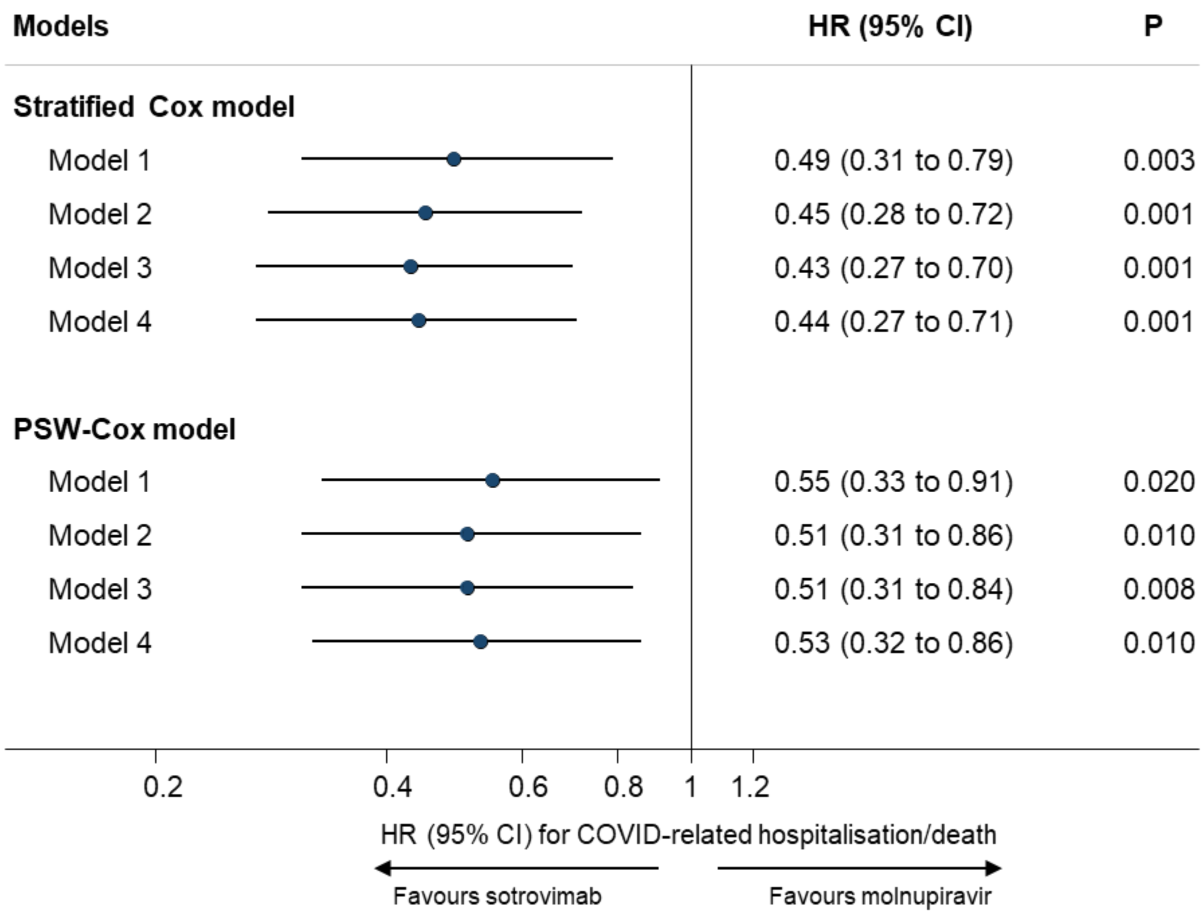

Supplementary Figure 3. Comparing risk of 28-day COVID-19 related hospitalisation/death between patients treated with sotrovimab vs. molnupiravir (Period 2).

Note: HR=hazard ratio; CI=confidence interval; PSW=propensity score weighting. Model 1 adjusted for age and sex; Model 2 additionally adjusted for ten high-risk cohort categories; Model 3 further adjusted for ethnicity, IMD quintiles, vaccination status, calendar week; and Model 4 additionally adjusted for BMI category, diabetes, hypertension, chronic cardiac and respiratory diseases.

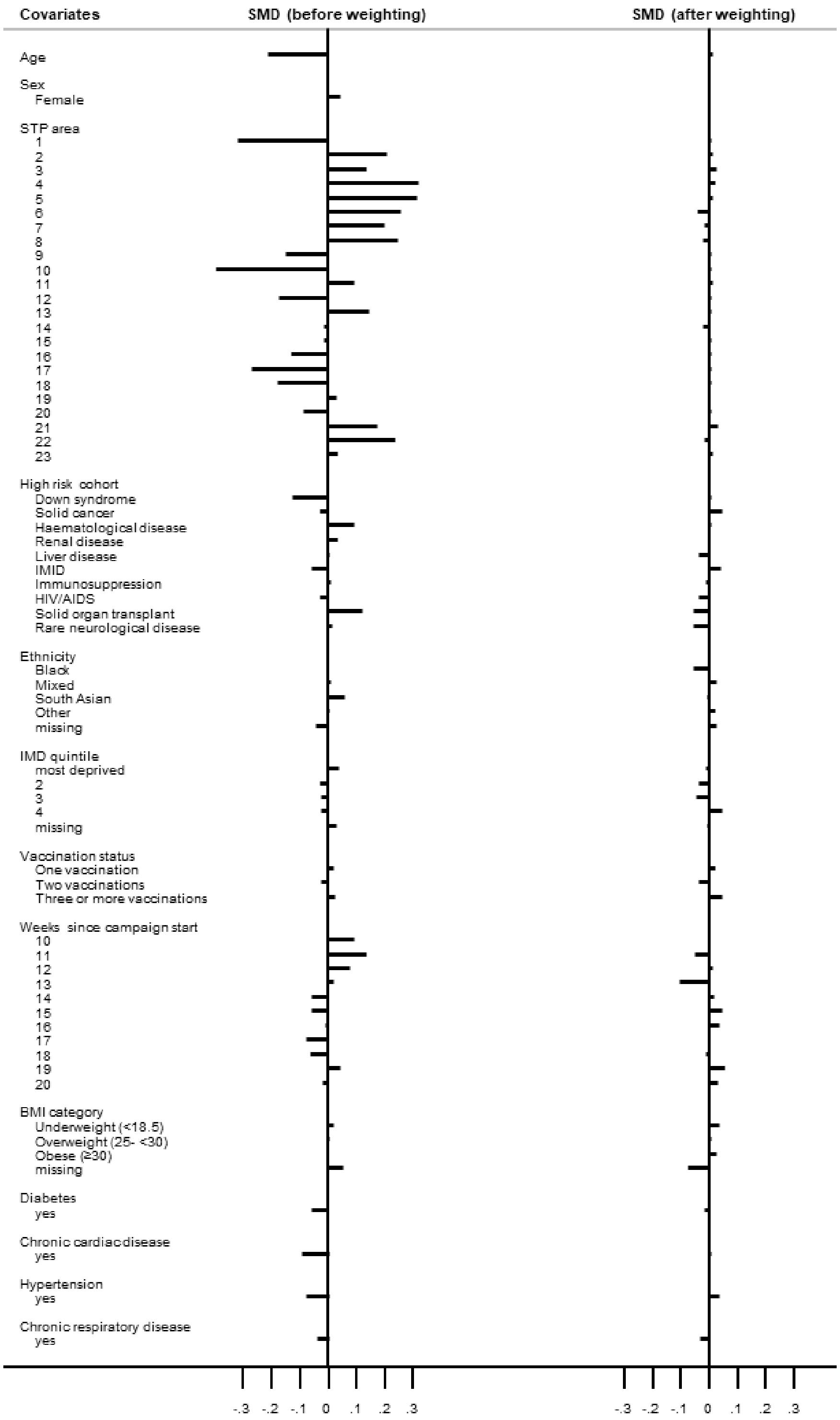

Supplementary Figure 4. Balance check of baseline covariates between treatment groups before and after propensity score weighting (Period 2).

Note: SMD=standardised mean difference; STP=Sustainability Transformation Partnerships; IMID=Immune-Mediated Inflammatory Diseases; IMD=Index of Multiple Deprivation; BMI=body mass index.
